# Acoustic Fabry–Perot Resonance Detector for Passive Acoustic Thermometry and Sound Source Localization

**DOI:** 10.3390/s25082445

**Published:** 2025-04-12

**Authors:** Yan Yue, Zhifei Dong, Zhi-mei Qi

**Affiliations:** 1 State Key Laboratory of Transducer Technology, Aerospace Information Research Institute, Chinese Academy of Sciences, Beijing 100190, China; yy853140508@163.com (Y.Y.); dzfb014112@163.com (Z.D.); 2 School of Electronic, Electrical, and Communication Engineering, University of Chinese Academy of Sciences, Beijing 100049, China; 3 School of Optoelectronics, University of Chinese Academy of Sciences, Beijing 100049, China

**Keywords:** acoustic Fabry-Perot resonance detector (AFPRD), acoustic temperature measurement (ATM), sound source localization (SSL)

## Abstract

Acoustic temperature measurement (ATM) and sound source localization (SSL) are two important applications of acoustic sensors. The development of novel acoustic sensors capable of both ATM and SSL is an innovative research topic with great interest. In this work, an acoustic Fabry-Perot resonance detector (AFPRD) and its cross-shaped array were designed and fabricated, and the passive ATM function of the AFPRD and the SSL capability of the AFPRD array were simulated and experimentally verified. The AFPRD consists of an acoustic waveguide and a microphone with its head inserted into the waveguide, which can significantly enhance the microphone’s sensitivity via the FP resonance effect. As a result, the frequency response curve of AFPRD can be easily measured using weak ambient white noise. Based on the measured frequency response curve, the linear relationship between the resonant frequency and the resonant mode order of the AFPRD can be determined, the slope of which can be used to calculate the ambient sound velocity and air temperature. The AFPRD array was prepared by using four bent acoustic waveguides to expand the array aperture, which combined with the multiple signal classification (MUSIC) algorithm can be used for distant multi-target localization. The SSL accuracy can be improved by substituting the sound speed measured in real time into the MUSIC algorithm. The AFPRD’s passive ATM function was verified in an anechoic room with white noise as low as 17 dB, and the ATM accuracy reached 0.4 °C. The SSL function of the AFPRD array was demonstrated in the outdoor environment, and the SSL error of the acoustic target with a sound pressure of 35 mPa was less than 1.2°. The findings open up a new avenue for the development of multifunctional acoustic detection devices and systems.

## 1. Introduction

Acoustic waveguides have unique advantages in acoustic wave transmission and control, because the transmission behavior of the internal acoustic waves is significantly affected by the geometric structure, material properties and boundary conditions of the waveguide. With the help of these mechanisms, the propagation efficiency of acoustic wave energy can be optimized, the transmission stability of signals can be enhanced, and new acoustic devices can be designed. They have been widely used in fields such as medicine [1], noise control [2] and voice transmission [3]. However, in the fields of ATM and SSL, the application of acoustic waveguides is not sufficient.

ATM technology has gradually emerged due to its advantages such as high accuracy, wide measurement range, and strong anti-interference ability [4,5,6,7]. It is very suitable for gas temperature measurement in complex or dangerous environments, such as high-temperature boiler furnaces [8] and inside power plants [9]. The non-contact feature can prevent contamination of the device itself in the biomedical field, and can be used to monitor the temperature of drugs and biological samples in refrigerated or constant temperature equipment [10]. ATM devices based on traditional acoustic resonant cavities are usually closed structures [11,12]. Sound waves reflect and interfere in the cavity to form standing waves, thereby generating a resonant effect at a specific frequency. The temperature of the medium is measured by the influence of temperature changes on the resonance effect. This structure can effectively isolate external noise and obtain extremely high measurement accuracy. Feng [13] used microwave resonant frequency to accurately measure the length of a cylindrical copper cavity and measured the Boltzmann constant by the gas temperature method. Underwood [14] used a circular quasi-spherical resonator to measure the internal neon temperature to evaluate the international temperature scale ITS-90. However, the inability to exchange factors such as gas or temperature with the external environment limits its flexibility and adaptability in practical applications outside the laboratory.

SSL technology has been widely used in the fields of industry [15], national defense security [16], and smart devices [17], but it still faces challenges in long distances [18], low signal-to-noise ratios and complex environments. Traditional SSL methods are limited by problems such as insufficient sensor sensitivity [19], environmental noise interference [20], and array size limitations [21]. Tang [22] used acoustic waveguides to design artificial periodic structures, controlled the amplitude of sound waves through high transmittance in specific frequency bands and flexibly changed the unit size to adjust the phase, thereby achieving amplitude and phase modulation of sound waves. Rutsch [23] designed an ultrasonically coupled phased array using acoustic waveguides. By estimating and compensating for additional time delays, beamforming can be achieved without sacrificing sound pressure level and enabling rapid assembly. Although the traditional acoustic phased array based on acoustic waveguide can improve the spatial resolution and signal processing capability by selectively controlling the sound waves of different frequencies, it still does not solve the problem of the overly large array.

For this reason, we choose a semi-closed tubular acoustic waveguide as the structure of the acoustic resonant chamber. One end of the acoustic waveguide is a microphone diaphragm that acts as a hard boundary, and the other end is connected to the outside space to form a semi-closed open system. The working principle of this structure is based on the propagation, reflection and interference of inside sound waves. When the sound wave enters the closed end of the waveguide, it is reflected due to its high acoustic impedance. The open boundary at the other end allows the sound wave to exchange with the outside environment, forming a soft boundary. This hard-soft boundary configuration allows the sound wave to form a standing wave mode of the specific frequency inside the waveguide while being able to exchange gas with the outside world. Its working principle is similar to the interference effect of light waves in an optical Fabry-Perot interferometer [24], so it is called an acoustic Fabry–Perot resonator detector (AFPRD).

The AFPRD with an open port allows for the rapid exchange of internal and external gas, which is beneficial to the measurement of ambient gas temperature, and its simple structure is also conducive to practical production. By applying the AFPRD based on a bent acoustic waveguide to the array acoustic positioning system, its sensitivity enhancement characteristics of specific frequencies can improve the positioning performance of low signal-to-noise ratio sound sources. The directional resonant gain effectively suppresses noise signals from non-target directions. The bent acoustic waveguide can flexibly expand the array element position while the acoustic sensor is centrally fixed, avoiding the array size being too large. This study analyzes the acoustic characteristics of AFPRD through finite element simulation and preliminary experiments, verifies the feasibility of applying it to ATM and SSL, and provides theoretical support for further practical application.

## 2. Principles of ATM and SSL

### 2.1. AFPRD Model and Resonance Principle

**Figure 1:**
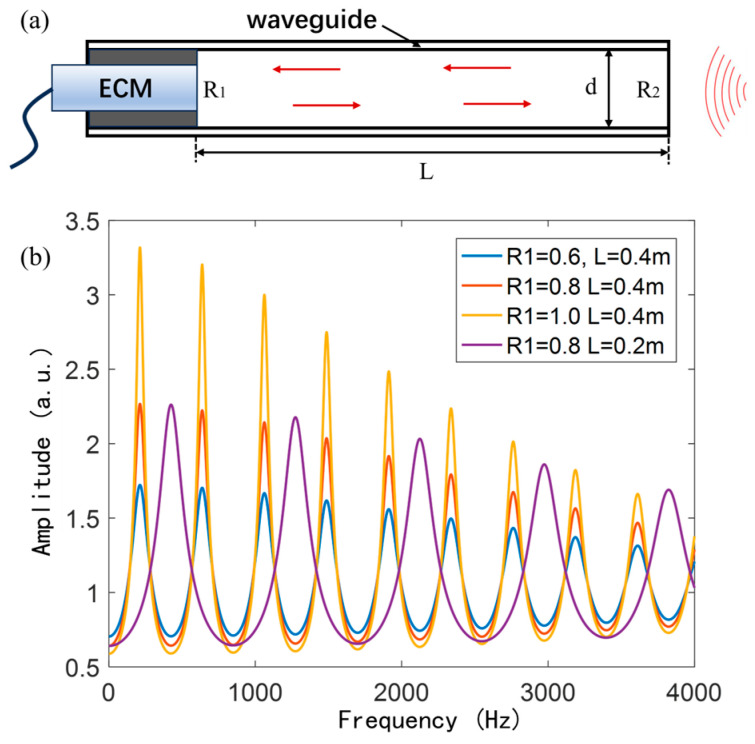
(**a**) AFPRD structure designed in this work; (**b**) Simulated frequency response curves of the AFPRD with different acoustic reflectivities of the endface and different lengths of the FP cavity.

As shown in Figure 1a, the AFPRD model consists of a resonant cavity with a length L, where R_1_ represents the reflectivity of the electret condenser microphone (ECM) diaphragm, and R_2_ denotes the reflectivity of the open end. The sound wave enters the waveguide from the open end and undergoes multiple reflections. The sound wave amplitude at the diaphragm can be described by reflectivity, amplitude attenuation, phase change, and amplitude recursive relationship. The amplitude of the incident wave is $P_{0}$, and the amplitude after propagation attenuation is $P_{0}e^{-\alpha L}$, where α is the attenuation coefficient. After reflection, the phase changes by $\phi_{1}$, so the reflected wave is as follows:

(1) $$P_{1}=R_{1}P_{0}e^{-\alpha L}e^{i\phi_{1}}$$

When the sound wave is reflected at the hard boundary, an additional phase shift of π will be generated. Adding the phase shift caused by the waveguide length L, $\phi_{1}=\pi +\frac{2\pi fL}{c}$. The second reflection is the reflection at the soft boundary, and the reflected wave and the incident wave keep the same phase. Then $\phi_{2}=\frac{2\pi fL}{c}$. The wave after reflection is as follows:

(2) $$P_{2}=R_{2}P_{1}e^{-\alpha L}e^{i\phi_{2}}={R_{2}R}_{1}P_{0}{\left(e^{-\alpha L}\right)}^{2}e^{i{(\phi }_{1}{+\phi }_{2})}$$

Then the final amplitude at the diaphragm is obtained by superimposing the results after each reflection. The attenuation coefficient α usually depends on the frequency of the sound wave and can be expressed as $\alpha =kf^{2}$. The sound pressure at the diaphragm can be expressed as follows:

(3) $$P\left(t\right)=e^{-\alpha L}e^{i\frac{2\pi fL}{c}}\left(P_{0}+P_{2}+P_{4}+\ldots +P_{2n}\right)\;=e^{-\alpha L}e^{i\frac{2\pi fL}{c}}P_{0}\left[1+{R_{2}R}_{1}e^{-2\alpha L+i{(\phi }_{1}{+\phi }_{2})}+\ldots +{\left({R_{2}R}_{1}e^{-2\alpha L+i{(\phi }_{1}{+\phi }_{2})}\right)}^{n}\right]\;=\frac{e^{-kf^{2}L+i\frac{2\pi fL}{c}}P_{0}}{1+{R_{2}R}_{1}e^{-2kf^{2}L+i\frac{4\pi fL}{c}}}$$

The amplitude of the sound pressure:

(4) $$P=\frac{P_{0}}{\sqrt{1+{\left(R_{1}R_{2}\right)}^{2}e^{-4kf^{2}L}+2\left(R_{1}R_{2}\right)e^{-2kf^{2}L}\cos\left(\frac{4\pi fL}{c}\right)}}$$

It is seen from Equation (4) that when $\cos\left(\frac{4\pi fL}{c}\right)=-1$, the sound pressure amplitude reaches the maximum value, indicating that this frequency is the resonant frequency $f_{n}$.

(5) $$f_{n}=\frac{\left(2n+1\right)c}{4L},with\;n=1,\;2,\;3,\;\cdot \cdot \cdot$$

where *n* represents the resonant mode order. As shown in Figure 1b, it is a numerical simulation of the frequency response curve under different microphone diaphragm reflectivity and cavity length. It can be seen that the higher the diaphragm reflectivity and the closer it is to the ideal rigid boundary, the greater the resonant amplification and the better the suppression effect of the non-resonant frequency. The resonant frequency is related to the AFPRD cavity length. The longer the cavity, the more resonances it can accommodate.

### 2.2. Sound Velocity and Temperature Measurement Based on AFPRD

In a gas medium, the speed of sound is related to the compressibility and density of the gas. In addition, the propagation speed of sound waves in a waveguide is much greater than the heat transfer speed, making a single temperature measurement approximately an adiabatic process. Combined with the Clapeyron equation of the ideal gas model, the speed of sound can be converted into a single-valued function of temperature.

(6) $$c=\sqrt{\frac{dP}{d\rho }}=\sqrt{\frac{\gamma P}{\rho }}=\sqrt{\frac{\gamma RT}{M}}$$

Therefore, the AFPRD resonant frequency can be expressed in terms of temperature, as follows:

(7) $$f_{n}=\frac{\left(2n-1\right)c}{4L}=\frac{\left(2n-1\right)}{4L}\sqrt{\frac{\gamma RT}{M}}$$

where *R* is the gas molar constant, *M* is the gas molar mass, and *γ* is the adiabatic factor. This means that as long as the real-time measurement of the sound velocity is approximated as an adiabatic process, the sound velocity is only related to the temperature, thus avoiding the influence of air pressure and gas density under different altitude conditions. For dry air, the adiabatic index can be approximated to 1.4, and the molar mass is 28.96 g/mol. Taking into account the climate differences in the actual measurement environment, the parameters need to be corrected according to the environmental conditions. Humidity affects the molar mass and adiabatic index of the atmosphere.

(8) $$\gamma =\frac{\left(1-x\right)C_{p,dry}+{xC}_{p,water}}{\left(1-x\right)C_{v,dry}+{xC}_{v,water}}$$

(9) $$M=\left(1-x_{m}\right)M_{dry}+x_{m}M_{water}$$

where x is the mass fraction of water vapor in wet air, and $x_{m}$ is the molar mass fraction. The constant pressure, constant volume specific heat capacity and molar mass of dry air are $C_{p,dry}$, $C_{v,dry}$, and $M_{dry}$ respectively, and the constant pressure, constant volume–specific heat capacity and molar mass of water vapor are $C_{p,water}$, $C_{v,water}$ and $M_{water}$ respectively. As humidity increases, the adiabatic index and molar mass of actual air will further decrease.

**Figure 2:**
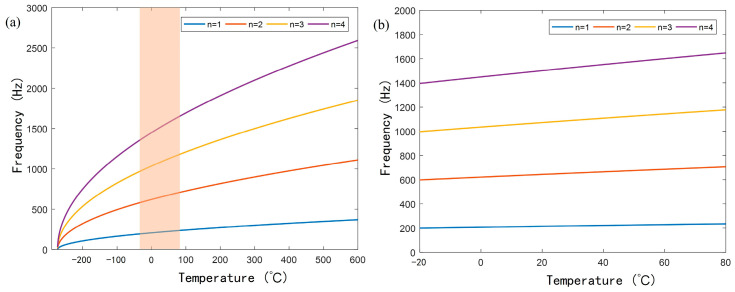
(**a**) The relationship between the first four resonant frequencies and temperature; (**b**) The linear change in resonant frequency with temperature in the range of −20 °C to 80 °C.

As shown in Figure 2a, each resonant frequency of the AFPRD has a corresponding functional relationship with temperature. Therefore, in theory, any resonant frequency can be used as a reference for temperature measurement. Since $f_{n}\propto \sqrt{T}$, the resonant frequency $f_{n}$ and temperature *T* show a nonlinear relationship. However, in actual temperature measurement applications, this nonlinear relationship has little effect on accuracy. On the one hand, as shown in Figure 2b, in the practical temperature range of −20 °C to 80 °C, the resonant frequencies of each order and the temperature can be approximated to be linearly related, and the coefficient of determination R^2^ of the fitting line exceeds 0.999. On the other hand, because the temperature measurement process is to measure the resonant frequency and infer the temperature in combination with the known system parameters, as long as these parameters can be accurately obtained and kept stable, the temperature can be solved through the known mathematical model.

### 2.3. AFPRD-Based Acoustic Localization Array

**Figure 3:**
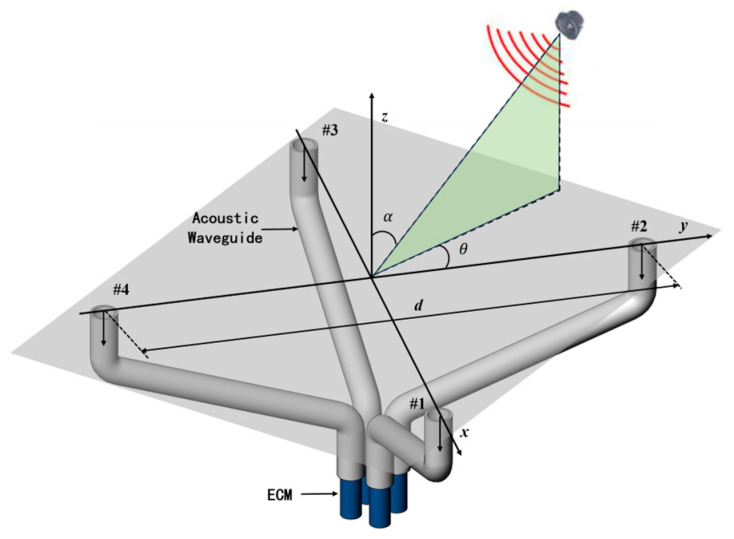
Schematic diagram of the AFPRD array for SSL.

AFPRD can not only be used to measure temperature, but its specific frequency enhancement effect and real-time response to sound speed can also be used for SSL. In particular, it has improved the emerging spatial spectrum-based sound localization algorithm. Multiple signal classification (MUSIC) is a type of spatial spectrum sound localization algorithm. In the far-field model, the signal array X received by the probe in the four-element cross array can be expressed as follows:

(10) X=X1X2X3X4=ej2πd1λ∗S∗1ejπdsin⁡θ−cos⁡θλsinαej2πdsin⁡θsinαejπdsin⁡θ+cos⁡θλsinα+Nk=ej2πd1λ∗S∗A+Nk

where *S* is the sound source signal, *A* is the direction matrix determined by the AFPRD array and it is a function of angular frequency (ω), azimuth (θ) and elevation angle (α), $N_{k}$ is the noise source, d is the array aperture, and *λ* is the wavelength of the sound wave and can be expressed as *λ* = *c*/*f*. The acoustic positioning array model based on AFPRD is shown in Figure 3. Compared with the slender cylindrical acoustic waveguide, the array aperture can be expanded by the bent acoustic waveguide. The position of the array element is determined by the position of the sound inlet rather than the ECMs, so that the ECM can be centrally managed while achieving a more flexible array arrangement. Of course, this is based on the premise that the curved acoustic waveguide has the same resonance characteristics. According to Formula (5), the AFPRD enables real-time measurement of the sound speed. Because the sound speed in the actual test environment is affected by factors such as temperature and humidity, the wavelength *λ* in Formula (10) can be calibrated in real time by sound speed measurement. The covariance matrix of the signal array *X* can be decomposed into the following eigenvalues:

(11) $$\begin{array}{c} \;R\left(\omega ,t\right)=\frac{1}{TN}\sum_{\tau =t}^{\tau =t+TN-1}X\left(\omega ,\tau \right)X^{*}\left(\omega ,\tau \right)=U_{s}\sum_{s}U_{s}^{H}+U_{n}\sum_{n}U_{n}^{H} \end{array}$$

where $U_{s}$ and $U_{n}$ are the signal subspace and noise subspace respectively, $U_{s}^{H}$ and $U_{n}^{H}$ denote the Hermitian transpose. The array spatial spectrum function is expressed as follows:

(12) $$P\left(\theta ,\alpha \right)=\frac{1}{∆\omega }\sum_{\omega_{L}}^{\omega_{H}}\frac{1}{{\left|A\left(\omega ,\theta ,\alpha \right)U_{n}\right|}^{2}}$$

where $A\left(\omega ,\theta ,\alpha \right)U_{n}$ indicates the orthogonality of the direction vector and the noise subspace. If the direction vector matches the sound source direction, then *A* will be approximately orthogonal to the noise subspace, so the inner product approaches 0, causing the $P\left(\theta ,\alpha \right)$ to approach infinity, and a peak to appear in the spatial spectrum. Based on the fact that the four sensor elements in the AFPRD array respond differently to the same sound source, when we scan all possible directions, we can obtain multiple values of $A\left(\omega ,\theta ,\alpha \right)$, and with these values a spatial spectrum $P\left(\theta ,\alpha \right)\;$is established, from which we can determine the direction of the sound source by searching the peak position.

**Figure 4:**
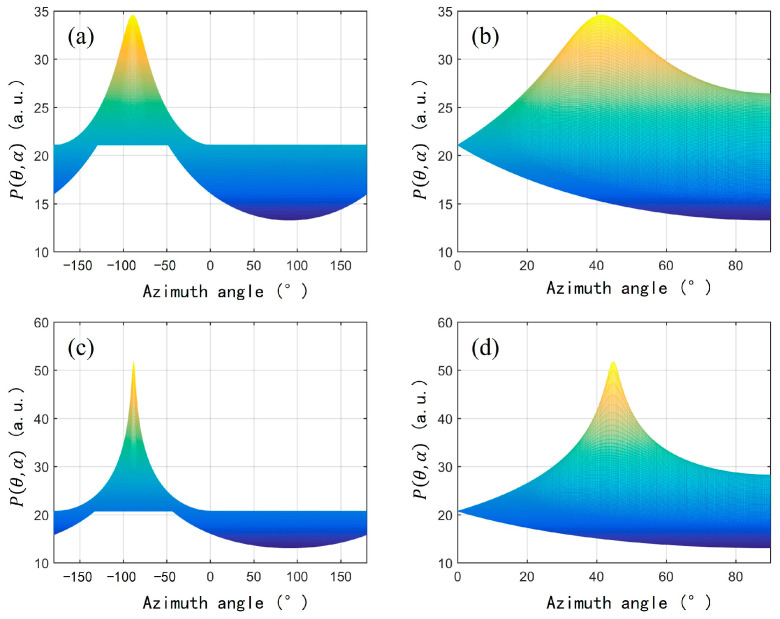
Spatial spectrum function *P*(*θ*, *α*) (**a**) SNR = 1, azimuth direction; (**b**) SNR = 1, elevation direction; (**c**) SNR = 10, azimuth direction; (**d**) SNR = 10, elevation direction.

Traditional MUSIC algorithms usually calculate spatial spectrum functions within a wide frequency band, and a large amount of data needs to be processed to improve algorithm performance. Based on the resonant characteristics of AFPRD, only limited frequency points near the center of the resonant frequency need to be selected. The frequency width of the resonance peak is generally 20–30 Hz, which significantly reduces the computational burden. And because the AFPRD has a selective amplification effect on the acoustic signal at the resonant frequency, the signal power received by the sensor is enhanced, the detection sensitivity is improved, and the background noise is not affected by this effect, so the overall signal-to-noise ratio is improved. As shown in Figure 4, under high signal-to-noise ratio conditions, the spatial spectrum peak is sharper, which is conducive to the identification of spatial angles and improves positioning accuracy and resolution.

## 3. Simulation Modeling and Principle Verification

### 3.1. Finite Element Simulation Model

**Figure 5:**
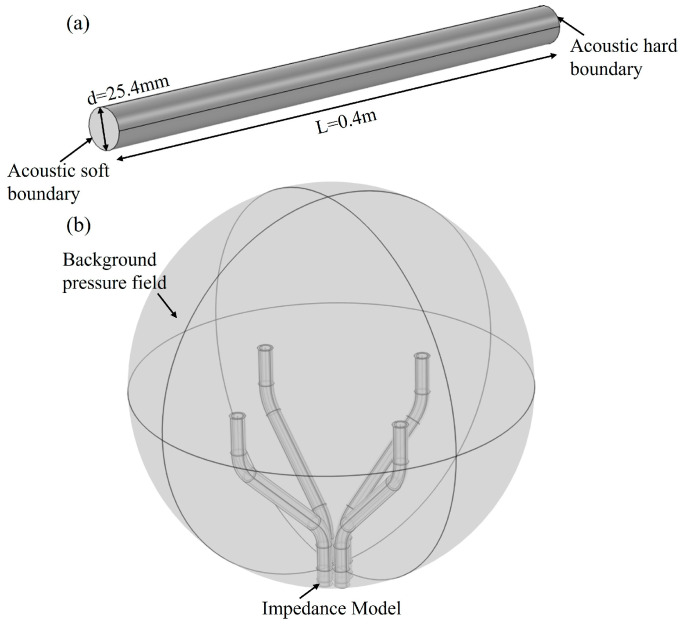
(**a**) Cylindrical AFPRD simulation model; (**b**) AFPRD array acoustic localization simulation model.

Finite element simulation of the characteristics of both AFPRD and its array was performed by Comsol, and the simulation models are shown in Figure 5. Figure 5a is a simulation of the interior of the acoustic waveguide in a cylindrical AFPRD. The acoustic waveguide is 0.4 m long and has an inner diameter of 25.4 mm. The open end is in direct contact with the air as a soft sound field boundary, and the side wall of the waveguide is a rigid hard sound field boundary. The sound outlet is a closed end that can simplify the hard sound field boundary or set the impedance according to the properties of the ECM diaphragm. Figure 5b is an AFPRD array based on a curved acoustic waveguide. The axial length is 0.24 m and the array aperture is 0.1 m. In order to simulate a real sound positioning scene, an air dielectric sphere is used to wrap the array to realize the background pressure field. The outer layer of the spherical shell is set as a perfect matching layer to absorb reflected waves. A plane wave is applied at a specific position to simulate the sound wave incidence. The wall of the acoustic waveguide is a hard sound field boundary, and the sound outlet is an impedance model to simulate the sensor diaphragm.

### 3.2. Thermoviscous Layer Effect

**Figure 6:**
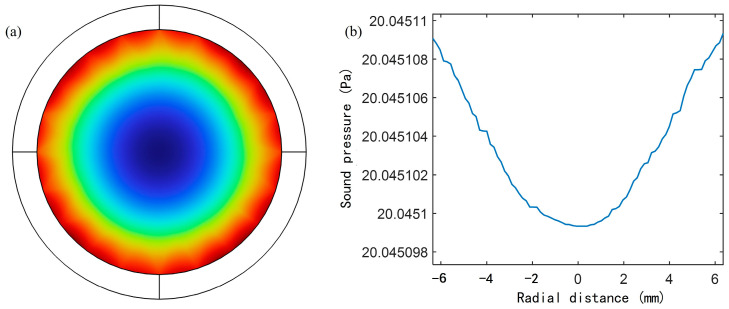
(**a**) Sound pressure distribution of AFPRD cross section under 1106 Hz sound wave; (**b**) Sound pressure distribution in the cross section of the acoustic waveguide.

The propagation of sound waves in the waveguide is not only limited by the geometric shape but also significantly affected by the thermal and viscous effects of the fluid. The thermoviscous layer is caused by the interaction between the temperature gradient and the pressure gradient of the fluid, which affects the sound velocity, attenuation, and sound field distribution in the waveguide. Due to the existence of thermoviscous effects, the propagation characteristics of sound waves often deviate from the ideal uniform plane wave assumption. For this reason, the inner wall of the acoustic waveguide in the simulation model is changed to a thermoviscous boundary, the gas flow in the cylinder and the heat exchange on the boundary surface are considered, and the pressure acoustics and thermoviscous acoustics physical fields are coupled. Since the thermoviscous layer is thin and changes greatly in the area close to the wall, the area near the cylindrical surface is finely meshed to ensure that the gradient changes can be accurately simulated. The simulation results are shown in Figure 6.

According to the sound pressure distribution results of the acoustic waveguide cross-section shown in Figure 6a, the sound pressure in the central area is slightly lower, while the sound pressure in the edge area is slightly higher. This is because the gas viscosity in the thermoviscous layer makes the gas flow velocity near the wall zero. In this case, the sound wave on the boundary will be locally reflected, and the superposition effect with the incident wave will cause the sound wave near the thermoviscous layer to be enhanced, thereby generating a larger sound pressure. The sound pressure sampling points are set along the diameter direction of the cross-section, and the results are shown in Figure 6b. Although the sound pressure distribution is uneven, the overall deviation is extremely small, and its relative error is less than one ten-thousandth, which is lower than the sensitivity threshold of general commercial microphones. Therefore, it can be considered that it will not have a significant impact on the measurement accuracy. In most cases, the effect of the thermoviscous layer is mainly concentrated in the area close to the wall. Therefore, in most areas of the acoustic waveguide, the propagation characteristics of the sound wave will not be significantly affected by the thermoviscous layer. In subsequent applications, the thickness of the thermoviscous layer needs to be fully considered. The acoustic waveguide with a certain redundancy in diameter can be designed so that the thermoviscous layer will not affect the diaphragm at the axis position.

### 3.3. ATM Based on Multi-Order Resonance Frequency

**Figure 7:**
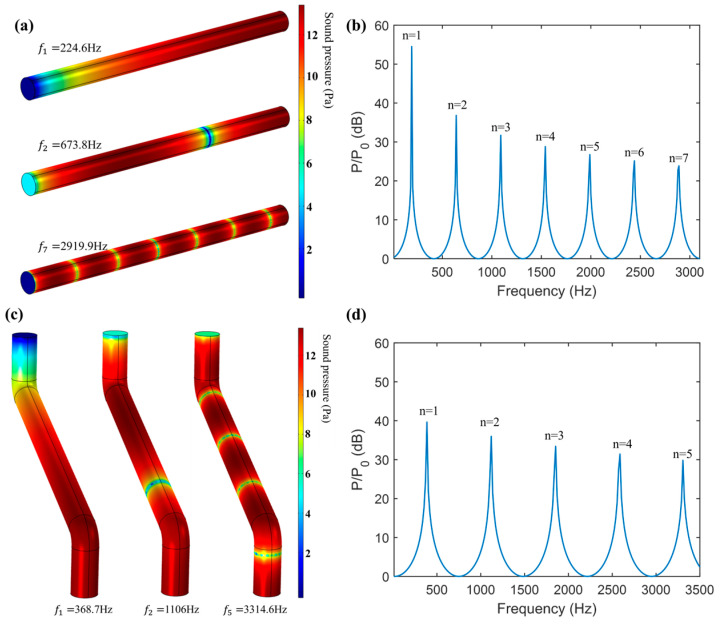
Simulation results of AFPRD: (**a**,**b**) the resonant modes and the frequency response curve for a 0.4 m long straight waveguide. (**c**,**d**) the resonant modes and the frequency response curve for a 0.24 m long bent waveguide.

Figure 7a,c shows the first two low-frequency resonant modes and high-frequency resonant modes of the internal sound field of the AFPRD with a straight waveguide and a bent waveguide. It can be seen that the resonant mode pattern of the AFPRD with the bent waveguide is consistent with that of the uniform cylinder, and the sound pressure at the sound outlet is the maximum value compared to the sound inlet, indicating that the AFPRD has achieved amplification at the resonant frequency. Through frequency response analysis, the frequency response curve of the AFPRD is shown in Figure 7b,d. There is a linear relationship between the multi-order resonance frequencies. Although the cavity lengths are different, they all match Formula (5). This shows that the cavity length plays a major role in the structural parameters of the AFPRD, and the inner diameter and bent structure of the acoustic waveguide do not affect the resonance frequency distribution. Compared with the straight waveguide, the bent waveguide only has a reduced amplification corresponding to the resonant frequency but still maintains an enhancement of more than 30 dB. The existence of a linear relationship makes the prediction of these high-order modes easier and more accurate. The consistency of the finite element simulation results with the numerical calculation results of Figure 1b also fully verifies the accuracy of the theoretical model. Especially for the open end of the cylindrical acoustic waveguide, the transmission of sound waves is allowed, and there is a certain relationship between the fluid velocity and pressure at the opening. Due to the fluidity of the gas, the open end can be approximated as a soft acoustic field boundary. This model well describes the transmission characteristics of sound waves at the open end.

However, according to Formula (5), a single resonant frequency can be used to infer the temperature. However, in actual measurement, in order to avoid the measurement error of a single-order resonant frequency, the measurement results of multiple-order resonant frequencies as shown in Figure 7b,d can be used to further infer the temperature through the linear relationship between the frequency and the order n. For multiple resonant modes of different orders, the frequency and the order show a linear growth relationship, and the slope S is expressed as follows:

(13) $$S=\frac{1}{2L}\sqrt{\frac{\gamma RT}{M}}$$

The multi-order resonance frequencies, slopes and temperature measurement results shown in Figure 7b,d are shown in Table 1. It can be seen that the shape of the acoustic waveguide has little effect on the ATM performance of the AFPRD. It can be found that there is a greater deviation in the reverse calculation of temperature through a single-order resonant frequency, and the measurement results of each order are inconsistent, while the temperature error obtained by the slope is the smallest.

**Figure 8:**
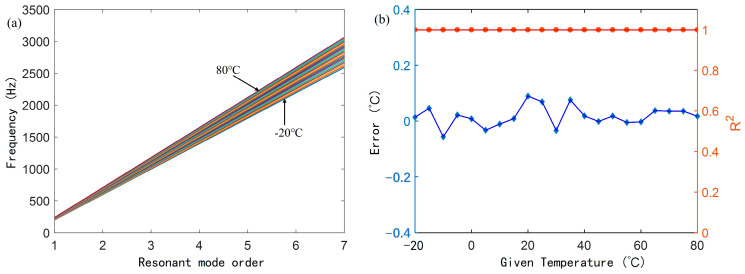
Temperature determination with the slope of the linear curve of the resonant frequency ($f_{n}$) versus the resonant mode order (*n*), (**a**) Simulated linear curves of $f_{n}$ versus *n* at different temperatures; (**b**) Temperature measurement error and fitting linearity based on the slope of the straight line.

With 5 °C intervals, the fitting straight lines of the first seven resonant frequencies and their orders of the simulated straight waveguide AFPRD under the conditions of −20 °C to 80 °C are shown. As shown in Figure 8a, it can be seen that the higher the temperature, the greater the slope of the fitting straight line. Figure 8b shows the error between the temperature inferred by the linear fitting slope and the simulated theoretical temperature. The maximum does not exceed 0.1 °C. And the linearity of each order of resonant frequency is good within the temperature range, and the determination coefficient R^2^ is greater than 0.999. The use of multi-order resonant frequency measurement can significantly improve the accuracy and stability of temperature inversion. Compared with single-order measurement, multi-order measurement can reduce single-point measurement errors and reduce the influence of random errors through regression analysis, thereby improving the accuracy of temperature estimation. The linear fitting of multi-order resonant frequencies can also compensate for the nonlinear influence between resonant frequency and temperature.

### 3.4. Performance Analysis of SSL Based on Bent Waveguide Array

**Figure 9:**
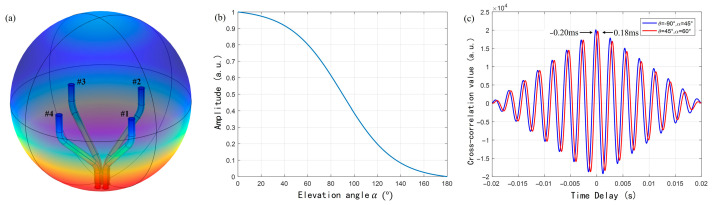
(**a**) Simulation model of the AFPRD array in an acoustic pressure field; (**b**) Normalized output sound pressure of AFPRD under different elevation angles; (**c**) Cross-correlation function of the time domain signals between sensor 1 and sensor 3 under two sound wave incident directions.

Figure 9a shows the sound pressure distribution inside the space when the simulated sound source is incident. The environment of the array is simulated by an air medium spherical shell, and a plane wave is applied at a specific position to simulate the actual sound wave incident. It can be found that the sound outlet position of the AFPRD still achieves resonance amplification. The air medium temperature is set to 48 °C, and the sound speed measured by the resonance frequency is 358.9 m/s. If the empirical value of the sound speed at room temperature of 343.3 m/s is still used in the positioning calculation, it will cause errors. The AFPRD array can measure the sound speed calibration algorithm in real time in an unknown environment. As shown in Figure 9b, the normalized output sound pressure distribution of the AFPRD at different sound source incident angles. The results show that the AFPRD can change the output sound intensity of sound waves incident from different directions so that it has a higher sound wave receiving efficiency in the direction of the tube axis and shows significant attenuation for sound waves incident at large angles. Within the range of 60° from the axis deviation, the normalized output sound pressure of the acoustic waveguide is always maintained above 0.8, indicating that the sound waves within this angle range can be efficiently coupled to the waveguide system. However, when the incident angle exceeds this range, the receiving efficiency drops rapidly, especially in the 180° direction, that is, the incident direction of the backward sound wave, where the sound pressure amplitude reaches the minimum, indicating that the AFPRD can effectively suppress environmental noise interference from the side and back.

The above simulation results show that the use of curved AFPRD for SSL can enhance specific frequencies and suppress environmental noise in non-target directions. However, it is still necessary to know whether it can accurately achieve the enlargement of the array aperture and whether the sound wave inside the bent waveguide is still a plane wave propagation. Therefore, in the simulation model, 360 Hz sound waves are incident at azimuth angle θ = −90°, elevation angle α = 45° and azimuth angle θ = 45°, elevation angle α = 60°, with a sound speed of 340 m/s. The time domain sound pressure of the AFPRD outlet at positions 1 and 3 in the array is sampled to solve the delay. The cross-correlation function is shown in Figure 9c, and the peak index values of the cross-correlation function are −0.20 ms and 0.18 ms, respectively. According to the structure, the sound inlet position is used as the array element position and the array aperture is 0.1 m. The theoretical delay differences are −0.206 ms and 0.1801 ms, respectively, and the simulation results are highly consistent with the theoretical model. This shows that the array can correctly enlarge the aperture, and the position of the AFPRD sound inlet determines that the array element position is independent of the sound outlet position. And the sound wave propagation inside the bent waveguide still follows the plane wave assumption, otherwise, the sound waves with different incident angles will bring different delays. This feature is crucial for subsequent SSL calculations because it ensures that the delay and phase measurements of different sensors can eliminate the phase shift added by the AFPRD cavity length through a simple differential operation.

## 4. Non-Ideal Factor Analysis

### 4.1. Limitation Analysis of High Temperature Gas

The implicit premise of temperature measurement by Formula (6) is that the gas temperature field is static, stable, and slowly varying in space. In the absence of convection, heat transfer in the gas is completed through molecular thermal motion, and the temperature at any point inside the waveguide is predictable. However, gas convection intensifies the energy transfer process at high temperatures. Because the local gas temperature fluctuates and is no longer stable, the sound velocity field becomes spatially inhomogeneous. In addition, the sound velocity will be affected by the airflow velocity component, introducing the Doppler effect. The Rayleigh number is a typical standard for natural convection activation:

(14) $$Ra=\frac{g\beta ∆TL^{3}}{\nu \alpha }$$

where g is the acceleration of gravity, *β* is the coefficient of thermal expansion, Δ*T* is the temperature difference between the upper and lower layers, *L* is the thickness of the gas layer, *ν* is the kinematic viscosity, *α* is the thermal diffusivity. Because the waveguide length is long when the temperature is greater than 100 °C, Ra > 1500. Natural convection in the gas begins to appear, and the temperature field and velocity field are no longer static and uniform.

In addition, the freedom of gas molecules gradually becomes active as the temperature changes. At high temperatures, *γ* tends to decrease because the increase in internal energy is mainly contributed by the vibration mode.

**Figure 10:**
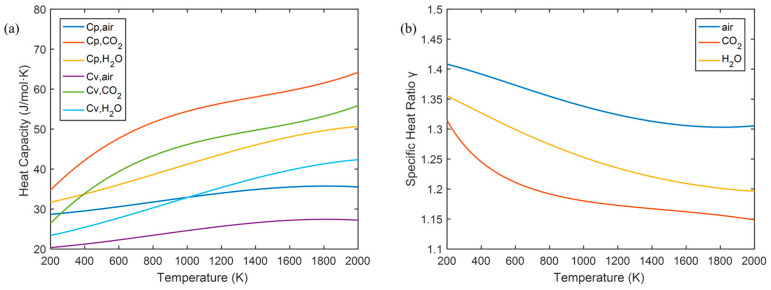
Temperature dependence of (**a**) the specific heat capacities *C_p_* and *C_v_*, and (**b**) the specific heat ratio *γ* for different gases.

Figure 10 shows the temperature variation curve of the specific heat ratio of different gases. It can be found that γ ≈ 1.4 for air at room temperature, and gradually decreases to about 1.35 as the temperature rises. The vibrational freedom of CO_2_ and H_2_O is easier to activate, and *γ* decreases more significantly. Therefore, for gases with a temperature greater than 200 K, a preliminary correction can be made using the formula:

(15) $$\gamma =\frac{\left(1-x_{H_{2}O}-x_{{CO}_{2}}\right)C_{p,air}+{x_{H_{2}O}C}_{p,H_{2}O}+{x_{{CO}_{2}}C}_{p,{CO}_{2}}}{\left(1-x_{H_{2}O}-x_{{CO}_{2}}\right)C_{v,air}+{x_{H_{2}O}C}_{v,H_{2}O}+{x_{{CO}_{2}}C}_{v,{CO}_{2}}}$$

Due to the fact that radiative heat transfer increases with the fourth power of temperature ($q_{rad}\propto T^{4}$), its influence becomes increasingly dominant at high temperatures. In this case, the local thermal state is no longer controlled by heat conduction and convection. The temperature of any point in the gas is affected not only by nearby heat sources, but also by distant strong radiation sources, and the temperature distribution is highly non-uniform and non-stable. Polyatomic gases (such as CO_2_, H_2_O) have strong infrared absorption and emission capabilities, and *γ* changes sharply after exciting the vibration mode. The propagation speed of sound waves in the waveguide is the “effective sound speed” on the path, not the sound speed at a certain point. Because of the spatial non-uniformity, the integral path is uncontrollable, and it is impossible to accurately infer the average or local temperature of the gas. Therefore, the AFPRD currently mainly works within 100 °C.

### 4.2. Effect of Acoustic Waveguide Structure Deviation

In practical applications, the geometric shape of the acoustic waveguide may deviate from the theoretical ideal cylindrical shape, and this deviation may affect the resonant frequency and amplification. Therefore, the degree of deviation of the acoustic waveguide shape from the ideal cylindrical shape and its impact on the resonant characteristics are studied through simulation. The AFPRD performance is simulated from two aspects: the uneven cross-sectional area of the acoustic waveguide and the incomplete closure of the diaphragm end.

**Figure 11:**
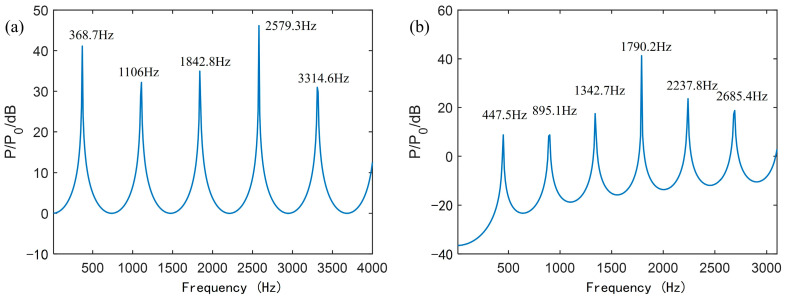
Frequency response curve of acoustic waveguide with (**a**) the non-uniform cross-section; (**b**) the closed-end leakage.

The cross-sectional diameter of the sound inlet of AFPRD is 21 mm, which gradually decreases to 20 mm along the axial direction. The frequency response curve is shown in Figure 11a. The amplitude at the resonant frequency is smaller than that of the uniform cylindrical AFPRD, and the resonant amplification of each order no longer follows the rule of the first-order maximum and then gradually decreases. This may be because the cross-sectional non-uniformity will cause the propagation speed of the sound wave to change in different areas. In the small cross-sectional area, the sound speed is larger, while in the large cross-sectional area, the sound speed is smaller. This will cause the bending or refraction of the sound wave propagation path, which will lead to a change in the effective length. According to Formula (5), the change in cavity length will cause a shift in the resonant frequency. Additionally, the non-uniform cross-sectional area may cause axial impedance discontinuities, reducing acoustic wave transmission efficiency and decreasing resonant amplification. Especially when the frequency is close to the resonant frequency, it may cause the mismatch of the standing wave mode, resulting in irregular resonant amplification of each order.

In the diaphragm cross-section of the closed end of the AFPRD, the section of the circular ring from the radius r = 8 mm to the boundary r = 10 mm is set as the soft sound field boundary directly connected to the outside air, and the area within the radius of 8 mm is the hard sound field boundary as the sensor diaphragm. This simulates the situation where the hard boundary is not completely closed. The frequency response curve result is shown in Figure 11b. Not only is the amplification at the resonant frequency significantly reduced, but the resonant frequencies of each order no longer follow the theoretical value distribution of the AFPRD. This is because when the closed end of the acoustic waveguide is not completely closed, the sound wave will partially leak out through the closed end, resulting in a weakening of the intensity of the reflected wave. This loss will reduce the resonant amplification. And the greater the degree of sound wave leakage, the weaker the interference effect, resulting in a shift in the resonant frequency. Therefore, the design of the closed end needs to be carefully considered to avoid excessive leakage and maintain the desired resonant characteristics.

## 5. Experimental Verification of ATM and SSL Functions

**Figure 12:**
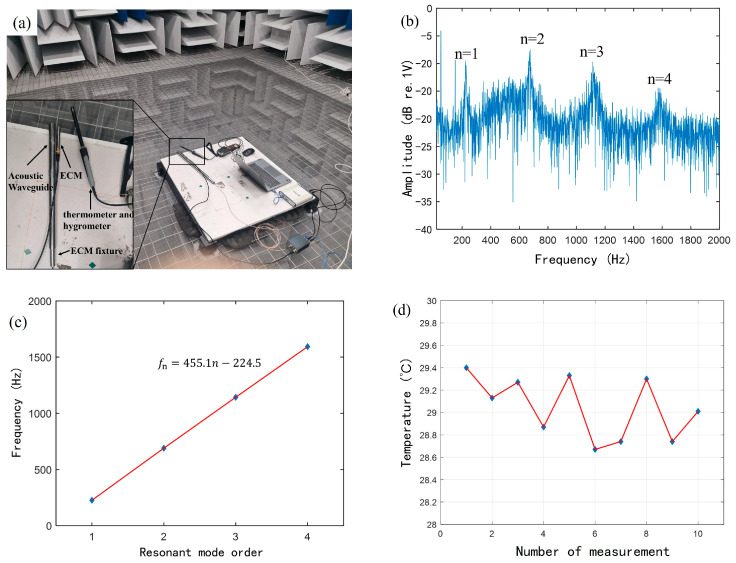
(**a**) Temperature measurement equipment in the anechoic room; (**b**) Frequency response curve under 17 dB background sound field; (**c**) Fitted line of the first four-order resonance frequencies; (**d**) Temperature results for 10 measurements.

In order to test whether the AFPRD can achieve internal multi-order resonance through environmental white noise, an ATM experiment was conducted in an anechoic room as shown in Figure 12a. The AFPRD was constructed by a stainless-steel tube acoustic waveguide and an ECM. The waveguide has a 2.54 cm diameter, 40 cm length, and 1 mm wall thickness. Combined with the microphone, the effective cavity length is 38.3 cm. Stainless steel has relatively higher rigidity and is less likely to deform in application. Its small thermal expansion coefficient can prevent the change in cavity length caused by thermal expansion. The front outer wall of the ECM fixture is fixed to the rear end of the acoustic waveguide through a thread, and a screw hole is opened on the side of the fixture to fix the microphone. The stability of the cavity length and the airtightness of the diaphragm end during measurement are guaranteed, and the changes in resonant characteristics caused by the above-mentioned structural errors are avoided.

The background noise level of the anechoic room calibrated by a separate ECM is 17 dB. At this time, the spectrum curve is shown in Figure 12b. Only the first four resonant peaks meet the signal-to-noise ratio requirements. Therefore, the slope of the first four resonant frequencies is selected for temperature calculation. As shown in Figure 12c, the slope of the fitting line is 445.1 Hz, and the temperature is calculated to be 29.15 °C according to Formula (13). If white noise is actively played to make the background sound field reach 23 dB, the first seven acoustic resonances can be achieved as shown in the simulation results. The ambient temperature is calibrated to 29.0 °C and the relative humidity is 29% by the thermometer and hygrometer. The result is shown in Figure 12d. The error of ten consecutive temperature measurements is less than 0.4 °C. In the extremely low sound pressure environment of the anechoic room, the AFPRD can still achieve the first four acoustic resonances. Although the error is 0.3 °C higher than the simulation, the first seven acoustic resonances can be achieved in actual environments such as outdoors, and the slope accuracy of the resonance frequency is higher. The AFPRD can output temperature data once per second, corresponding to a spectral resolution of 1 Hz. In high background noise environments, if the first seven resonant frequencies are utilized for temperature inversion, the spectral resolution can be reduced to 2 Hz, thereby improving the response speed to 0.5 s.

**Figure 13:**
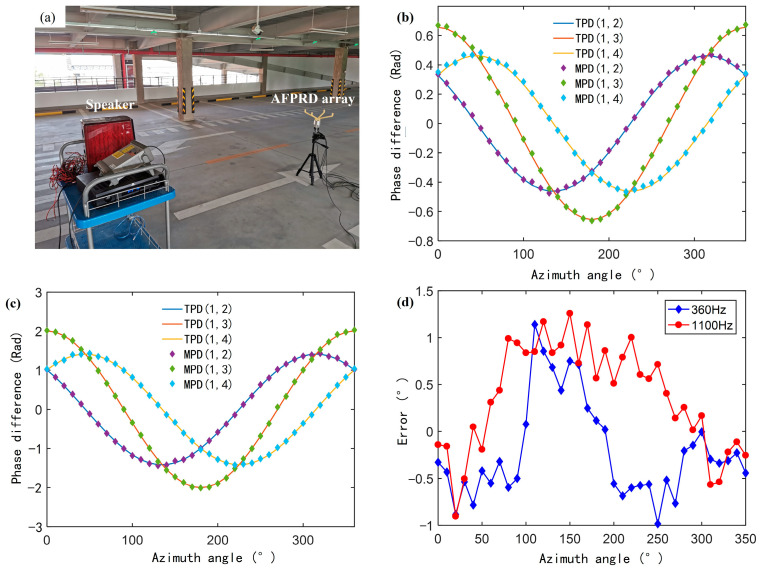
(**a**) Sound source localization test scene using the AFPRD array; (**b**) and (**c**) Measured phase differences (MPD) at two frequencies of 360 Hz and 1100 Hz between the two array elements of the AFPRD array and the corresponding simulated phase differences (TPD) (**d**) Azimuth angle measurement error.

In order to evaluate whether the AFPRD array can expand the array element spacing as expected and whether the bent acoustic waveguide will bring additional phase difference, an SSL test with a known sound source orientation was carried out as shown in Figure 13a.

Four ECMs were centrally installed in a specially designed cylindrical metal fixture, with an equivalent array diameter of 10 cm. The direction matrix A is constructed by the sound wave introduction position during the test. In order to verify its detection sensitivity to acoustic signals at the resonant frequency, weak single-frequency sounds with a sound pressure of 35 mPa and frequencies of 360 Hz and 1100 Hz were emitted by the speaker. The phase difference of each array element relative to the reference microphone (No. 1) was tested by rotating the rotating table from 0° to 360° in 10° steps, and compared with the theoretical calculation results of the directional matrix A. The ambient temperature calculated by the slope of the resonant frequency was 8 °C and the sound speed was 336.3 m/s, and subsequent calculations were based on this value. The experimental results are shown in Figure 13b,c. The extended array element spacing of the acoustic waveguide does not introduce significant phase estimation errors and ensures that the positioning accuracy will not be affected by the array structure adjustment in the subsequent sound source angle estimation process. As shown in Figure 13d, the azimuth angle estimation error of the single target sound source is less than ±1.2°, which proves the high accuracy and reliability of this method in the sound source localization task. For the dual-source case, we only need to sort the eigenvalues after the decomposition of Formula 11, and the last two eigenvalues correspond to the noise subspace. Although it has not been experimentally verified for moving sound sources, the algorithm we use has high real-time performance and has the potential to process moving sound sources.

## 6. Conclusions and Discussion

Both the passive ATM function of the AFPRD and the distant multitarget localization capability of the AFPRD array have been demonstrated theoretically and experimentally in this work. The findings show that: (1) The temperature measurement accuracy can be significantly improved by linear regression analysis of multi-order resonant frequencies. Under the simulation conditions of −20 °C to 80 °C, the maximum temperature error does not exceed 0.1 °C, which can effectively avoid the error accumulation caused by single-frequency measurement. In the anechoic room environment with a sound pressure of 17 dB, internal resonance can still be achieved and temperature measurement within an error range of 0.4 °C can be achieved through the first four resonant frequencies. (2) The bent AFPRD still maintains good resonance performance and can efficiently couple sound waves in different directions. It can reduce errors by calibrating the positioning algorithm through real-time measurement of sound velocity and temperature, and further improve accuracy through high resonant amplification and suppression of back noise. Cross-correlation function analysis and SSL experiments show that the bent structure can correctly enlarge the array aperture and can localize the sound source of weak sound signals of 35 mPa with an error of less than 1.2°. (3) The thermoviscous effect has little influence on the propagation characteristics of the acoustic waveguide. Through thermoviscous simulation, it is found that the thermoviscous layer has only a small effect on the sound pressure distribution in the area close to the wall. Its relative error is less than one ten-thousandth, and its influence can be ignored in the test phase. (4) Compared with the cross-section of non-uniform sound waves, the leakage effect significantly reduces the resonant amplification and causes a larger shift in the resonant frequency. Therefore, the geometric design of the acoustic waveguide and the optimization of the closed end are crucial to maintaining good resonant characteristics.

This method has good scalability and integrability. The acoustic sensor can be miniaturized into an on-chip structure through MEMS technology, and then the acoustic waveguide is extended as the array element of the array to form an on-chip array system. However, for underwater acoustic detection, the use of traditional condenser microphones is limited, and the thermoviscous boundary layer effect in water is more significant, which may cause greater sound loss in the waveguide. Therefore, it is necessary to consider using underwater special waveguide materials and modifying the sensor.

In summary, this study provides a theoretical and experimental demonstration of the outstanding ATM and SSL capabilities of the AFPRD-based acoustic system and is conducive to promoting its practical application in complex environments.

## Figures and Tables

**Table 1:** The first seven resonant frequencies (*f*_R_) of the AFPRD measured at 48 °C and the derived temperatures (T).

Mode Order	L = 0.4 m Straight Waveguide		L = 0.24 m Bent Waveguide	
	*f*_R_ (Hz)	T (°C)	*f*_R_ (Hz)	T (°C)
1	224.61	49.31	368.71	48.18
2	678.83	48.36	1106.0	48.11
3	1123.0	48.17	1842.8	47.92
4	1572.3	48.09	2579.3	47.77
5	2021.5	48.36	3314.6	47.45
6	2470.7	48.27		
7	2919.9	48.21		
Slope	449.2	48.08	736.51	47.85

## Data Availability

To access the data, please contact the corresponding author.
